# The “graying” of infertility services: an impending revolution nobody is ready for

**DOI:** 10.1186/1477-7827-12-63

**Published:** 2014-07-09

**Authors:** Norbert Gleicher, Vitaly A Kushnir, Andrea Weghofer, David H Barad

**Affiliations:** 1 Center for Human Reproduction, New York, NY 10021, USA; 2 Foundation for Reproductive Medicine, New York, NY 10021, USA; 3 Department of Gynecologic Endocrinology and Reproductive Medicine, Medical University Vienna, Vienna 1090, Austria

**Keywords:** Infertility, Advanced age, Infertility treatments, Age limitations, Clinical consequences, Societal consequences

## Abstract

**Background:**

As demand for infertility services by older women continues to grow, because achievable in vitro fertilization (IVF) outcomes are widely underestimated, most fertility centers do not offer maximal treatment options with use of autologous oocytes. Limited data suggest that clinical IVF outcomes in excess of what the *American Society for Reproductive Medicine* (ASRM) considers “futile” can, likely, be achieved up to at least age 45 years.

**Methods:**

In an attempt to point out an evolving demographic trend in IVF, we here report our center’s IVF data for 2010-2012 and national U.S. data for 1997-2010. Though our center’s data are representative of only one IVF center’s patients, they, likely, are unique since they probably represent the most adversely selected IVF patient population ever reported and, thus, are predictive of future demographic trends. In addition we performed a systematic review of the literature on the subject based on *PubMed*, *Medline* and *Google Scholar* searches till year-end 2013. The literature search was performed using key words and phrases relevant to fertility treatments in older women.

**Results:**

As demonstrated by our center’s patient demographics and national U.S. data, IVF centers are destined to treat increasingly adversely selected patients. Despite our center’s already extremely adversely selected patient population, age-specific IVF cycle outcomes in women above age 40 years, nevertheless, exceeded criteria for “futility” by the ASRM and widely quoted outcome expectations in the literature for patient ages. Age 43 discriminates between better and poorer clinical pregnancy and live birth rates.

**Conclusions:**

“Graying” of the infertility populations in the developed world, a problem with potentially far-reaching medical and societal consequences, has so far been only insufficiently addressed in the literature. As women’s postmenopausal life spans already exceed postmenarcheal life spans at the start of the 20^th^ century, the “graying” of infertility services can be expected to further accelerate, no longer as in recent decades bringing only women in their 40s into maternity wards but also women in their 50s and 60s. Medicine and society better get ready for this revolution.

## Background

Women above age 40 in the United States (U.S.) now represent the proportionally fastest growing age group having children [1,2]. Potential medical and societal consequences have found little attention in the medical literature, even though a recent study once again offered convincing evidence for the importance of age as a predictor of failure to achieve live birth [3].

Because favorable patients now usually conceive relatively quickly with in vitro fertilization (IVF), unfavorable patients accumulate disproportionally in IVF centers. This trend is further aggravated by above noted new reality in the U.S. that older and older women are trying to conceive.

Since our center for at least five years has been serving primarily as a “center of last resort” for patients who previously have failed IVF cycles elsewhere, our center experience, likely, is predictive of where the practice of IVF is destined to go over the coming decades.

We, therefore, reviewed in addition to national U.S. trends, our center’s 2010-2012 IVF outcome data, obtained in a uniquely adversely selected patient population, to assess outcome expectations, considering current practice patterns, for women above age 40 years. We, in addition, conducted a systemic search of the published literature on this subject.

This presentation, therefore, primarily is not meant as a presentation of original data but as a review of preliminary existing data, which may point towards where IVF practice, likely, is destined to go over the next two decades.

## Methods

### IVF cycle outcomes

Our center’s IVF cycles, under a federally mandated law, are annually reported to the *Centers for Disease Control* (CDC) and voluntarily to the *American Society for Reproductive Medicine* (ASRM)/*Society for Assisted Reproductive Technologies* (SART).

In addition, every cycle is entered into the center’s anonymized electronic research database, which served as one source for here reported center data for 2012 IVF cycles, together with the center’s annual reports to CDC and ASRM/SART. 2013 outcome data were not yet complete by the time of this report.

### Our center’s patient population

**Figure 1:**
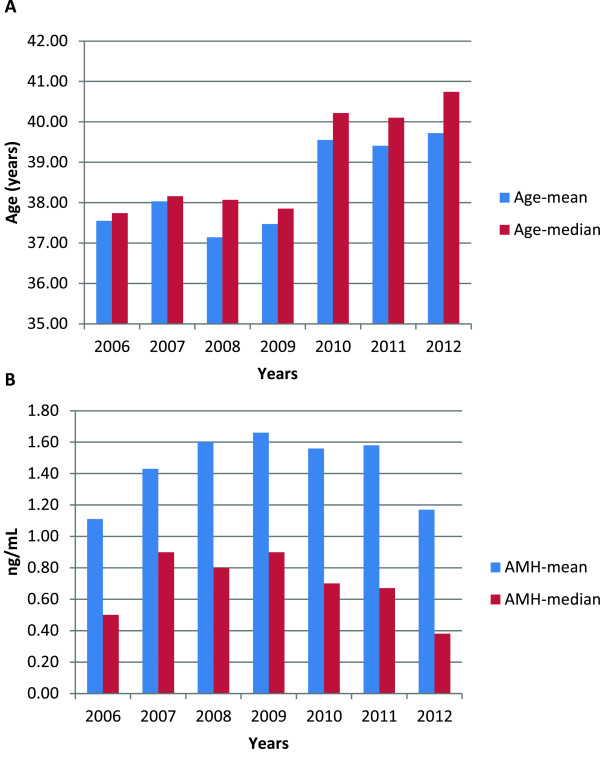
**Our center’s patient characteristics, years 2006-2012. A**. Mean and median age of patients in years 2006-2012 (in years). **B**. Mean and median AMH values (ng/mL) in years 2006-2012.

Our center’s IVF experience should be of special interest because of the patient population in which these outcomes were achieved. Based on review of 2010 and 2011 published CDC and ASRM/SART outcome reporting data, our center, proportionally, has been serving the largest percentage of women above age 42 years amongst all reporting ART centers in the U.S. Other adverse selection parameters for our center’s patient population are, however, not public since they are not reported to either CDC and/or ASRM/SART. Figures 1A and B demonstrate that our center’s patient population, indeed, does likely reflect the most adverse selection criteria of any reported IVF center in the U.S., if not worldwide.Figure 1A demonstrated mean and median ages of our center’s IVF patient population between 2006-2012. As the figure demonstrates, starting in 2010, our center experienced a significant increase in patient age, which even before already demonstrated a median population age around 38 years. Since 2010 the median age has, however, further increased to above age 40, with 2012 data suggesting that median age may soon reach age 41.

The “graying” of the center’s patient population is, however, not only demonstrated by increasing age. As Figure 1B demonstrates, concomitantly, the patients’ anti-Müllerian hormone (AMH) levels at presentation significantly declined, indicating increasingly poor functional ovarian reserve (FOR) of treated patients. Before 2010, median AMH values hovered around 0.80 ng/mL, already considered below favorable outcome levels for women with low FOR [4]; yet, starting in 2010, median AMH levels progressively decreased, reaching a nadir in 2012 below 0.40 ng/mL, with preliminary 2013 data (not shown) suggesting further declines.

Concomitantly, in our center’s 2012 annual CDC/SART submissions, 13.8% of all fresh IVF cycles occurred in women at ages 41-42 years, 20.6% at ages 43-44 years and 12.6% in women above age 46 years. This means that 47.0% of all fresh cycles involved women above age 41 and 33.2% of cycle women above age 43 years.

Considering these demographic data, it is not surprising that only 10.1% of fresh cycles were followed up by a frozen-thawed cycle during 2012 since women of advanced age and with low FOR rarely produce enough oocytes/embryos for subsequent thaw cycles. Yet, despite performance of so many fresh IVF cycles in significantly aged women, still, in addition, 18.3% of all fresh cycles were donor egg cycles.

Further documentation of our center’s adverse patient selection is the fact that between 2010-2012 in each year over 85% of newly presenting patients to our center had previously been in infertility treatments elsewhere and had failed at least one IVF cycle attempt. Most of these patients, indeed, had failed multiple IVF cycles, often at a number of different centers. In addition, the number of new long-distance patients, defined as patients from outside the larger New York City Tristate area, has been persistently increasing over the last five years, and in 2012 for the first time exceeded 60% of the centers total new patient population. Approximately two-third of long-distance patients come from the rest of the U.S. and Canada, and the rest from overseas.

Here presented data demonstrate that our center’s current population during 2012 in more than half of all cases was of very advanced female age and/or suffered from very low FOR. While such extreme patient characteristics, currently, are not yet the norm at other IVF centers, trends in developed countries go into the same direction, as young and uncomplicated IVF patients quickly conceive, while older and more poor prognosis patients accumulate.

### Systematic literature review

We searched *PubMed*, *MEDLINE®* and *Google Scholar* databases for multiple key words and phrases, referring to < fertility > and < fertility treatments > in < older women > or at < advanced age>, including specific end points, like < spontaneous pregnancies>, <pregnancy rates>, <in vitro fertilization (IVF)>, <intrauterine insemination (IUI)>, <miscarriages>, <pregnancy loss>, <aneuploidy>, <medical complications of pregnancy>, <age related medical complications of pregnancy>, etc.

Data from the literature were initially extracted by one author (NG) and then reviewed by the three other authors (VAK, AW, DHB).

This search failed to reveal even a single clinical trial addressing fertility treatments in “older” women (>age 40 - 42 years), and also did not demonstrate even a single review on the subject. Whatever limited data are available, are here presented.

### Institutional Review Board (IRB)

Our patients sign at initial consultation, as part of a universal HIPAA consent form, a statement that allows use of data from their medical records for reporting purposes to CDC, ASRM/SART and for research purposes, as long as their medical data remain confidential and their identity remains protected. Both conditions were met here. Since utilized data in here presented study only utilized anonymized statistical data sets, it did not require further IRB approval.

## Results

### Our center’s 2010-2012 IVF outcome data

The center’s annual 2010-2012 IVF outcome data are presented in Table 1. IVF outcomes are presented by “intent to treat”, which means with reference point cycle start. Considering how adversely selected here reported patients are, this is important to note. Moreover, as noted under “Materials and Methods”, a large majority of patients presenting to our center have failed prior IVF cycles elsewhere, often multiple cycles. Here reported 233 IVF cycles in most patients, therefore, followed prior failed IVF cycles elsewhere. In addition, a good number of here reported IVF cycles also represent repeat cycles at our center. IVF treatment outcomes, therefore, should at each age be significantly better than here reported if only first IVF cycles were to be considered. Such an analysis is, however, in here presented patient population not possible.

**Table 1:** Our center’s 2010 - 2012 age-specific clinical IVF outcome data by “intent to treat”*/** for women 40 years and older

**Age (years)**	**Live birth rate (%)**	**Clinical pregnancy rate (%) if different**
40	15.4	
41	42.9	
42	6.3	18.8
43	0.0	16.7
44	1.4	5.4
45	2.7	5.4
46-53	0.0	

*“Intent to treat” reflects denominator of per cycle start for each age group. A total of 233 IVF cycles are reported. Because of an ~20% cycle cancellation rate before embryo transfer, reports based on patients reaching embryo transfer of at least 1 embryo would demonstrate ~ 20% higher clinical pregnancy and live birth rates.

**Miscarriages reflect only established clinical pregnancies, confirmed by ultrasound by presence of at least one intrauterine gestational sac. Chemical and ectopic pregnancies are not considered in here presented data.

As Table 1 demonstrates, up to and inclusive of age 42 years, live birth rates are even in such an adversely selected patient population very respectable. Starting with age 43 years, there is a significant drop off in both clinical pregnancies and live births, as previously reported by our group for earlier years [5]. Above, and including age 46 years, pregnancy and delivery chances appear negligible, even though in 2013 our center established two, at this point ongoing clinical pregnancies, in women in their 46th year, which considering a small denominator, would reflect a respectable live birth rate if both pregnancies reach delivery (data not shown). Between 2010 – 2012 selected women up to age 53 were treated at our center, as Table 1 demonstrates.

It is also important to note that in women with still regular menses, at our center less than 20% fail to reach retrieval and/or transfer. This relative low cycle cancellation rate is based on our center’s policy to go to retrieval even with single follicles, unless patients object or patients are not already on maximal ovarian stimulation.

Likely, because of dehydroepiandrosterone (DHEA) supplementation, which at our center is routine in women with low FOR, miscarriage rates in even very adversely selected patients have in the past been only 15.1% [6]. Since publication of this study, the degree of adverse selection of patients at the center has, however, further increased (Figures 1A&B). During 2011-2012, miscarriage rates have, therefore, been in the 20.0-23.0% range. Table 1 demonstrates that miscarriages occurred primarily in the oldest patients. These data concur with our earlier report, which demonstrated that positive effects of DHEA on miscarriage rates progressively increase after age 35 but, of course, still increase with advancing female age [6].

Though overall modest, pregnancy rates obtained in a highly adversely selected patient population at our center, thus, clearly, at least up to and including age 42 but, likely, up to and including age 45 years, appear superior to the predominant opinions in the profession as to what is achievable in older women. Except for an outlier study by Ninimäki et al. [7], they also appear superior to studies we were able to discover in the literature on the subject (see below). They, of course, also exceed the definitions of the *Ethics Committee of the ASRM* for “futility” [8].

Our center’s IVF outcomes before 2012 have been widely published [5,6,9-15]. We, therefore, conclude that, based on clinical as well as ethical consideration, currently available data under appropriate informed consents do support a more proactive treatment of older women than is currently the prevailing practice in the U.S. and Europe. This argument appears further supported by the previously noted observation that even better outcomes than reported here can be expected in less adversely selected patients at older ages. Finally, unless the profession initiates more active treatment of older women, it is unlikely that maximal possible progress will be made in treating such women. In medicine, only practice “makes perfect”.

As important success of oocyte donation has become in offering maternity to older women, every egg donation, nevertheless, in at least some ways still represents treatment failures in a case of infertility. Rising numbers of oocyte donation cycles, as witnessed in many countries around the world, including the U.S., therefore, at least partially are a reflection of our profession’s failure in helping many older women to conceive with use of autologous oocytes.

### What our center’s IVF outcome data are based on

Progress in treating older women is continuous. For example, it only recently became known that at all ages low FOR is associated with low androgen levels [13-15], and that normal androgen levels (i.e., levels encountered in young women) are essential for early stages of follicle maturation [16,17]. We, therefore now pre-supplement older women with dehydroepiandrosterone (DHEA) in attempts to raise testosterone (T) levels [18]. Pregnancy chances in IVF depend on the degree of improvement in T levels after DHEA supplementation in such patients [13-15]. Indeed, we learned not to initiate IVF cycles in older women until T levels are in approximately the upper one-third of normal range or slightly above.

We also learned that so-called low-intensity cycles (“mini-IVF” or mild stimulation cycles) even in women with normal FOR produce inferior outcomes in comparison to standard IVF cycles [19,20]. Though a prospective clinical trial on this subject remains to be conducted, they, therefore, can be expected to produce even poorer outcomes in women with low FOR. In older women our center, therefore, practically universally only utilizes high dose gonadotropin stimulation in microdose-agonist cycles.

Our evolving approach to “older” ovaries is best demonstrated in a recently published study of 128 consecutive infertile women with extremely low FOR, defined by AMH values below 0.4 ng/m; many, indeed, had undetectable AMH levels. Their mean age was 40.8 ± 4.1 years, their mean baseline FSH was 15.7 ± 11.1 mIU/mL and their mean AMH was a remarkable 0.2 ± 0.1 ng/mL. Even this extremely adversely selected patient population still recorded a 7.9 percent clinical pregnancy (95% CI: 4.9-11.9%) per cycle, and a cumulative pregnancy rate of 15.6 percent (95% CI: 9.8-23.1%) in up to three consecutive IVF cycles. As one would expect, age 42 years significantly differentiated between better and poorer pregnancy (P = 0.013) and delivery chances (P = 0.036) [5].

Combining these data, published in 2011 and reflecting preceding years, with here presented, more recent outcomes, some interesting additional conclusions relating to patient age and FOR, as represented by AMH values, become possible: Likely the most important one is the relative irrelevance of even extremely low FOR (low AMH levels) up to and including age 42. Both of these studies demonstrate very clearly that, even with extremely low AMH or even undetectable levels, younger women and older women up to and inclusive of age 42 years, still have surprisingly good clinical pregnancy and live birth chances with IVF treatments. With and above age 43 years, a remarkable further drop off can be observed but serial cycles, likely, still will allow for decent cumulative returns up to and inclusive of at least age 45 years [5] and Table 1.

It again is important to reemphasize that here reported outcomes, because of adverse patient selection, likely, represent worst case scenarios, and even better outcomes can be expected in less adversely selected patients of same ages.

Utilization of high dosage gonadotropin stimulation has remained controversial. In women pretreated with DHEA, such an approach, however, does appear effective as larger oocyte numbers lead to more available euploid embryos for transfer [21]. This is an important observation since colleagues who reported that aneuploidy increases with higher gonadotropin dosages are, likely, correct [22,23]. They, however, overlook that increases in aneuploidy percentages are more than compensated by increases in absolute embryo numbers with DHEA supplementation. Despite increases in percentage of aneuploid embryos with high gonadotropin stimulations, properly selected patients, therefore, nevertheless end up with a net benefit in number of transferrable embryos [21]. Other investigators concur with these conclusions [24,25].

In older women, we also fail to understand the widely utilized practice of culturing embryos to blastocyst stage (days-5/6) since embryos at advanced female ages in the laboratory only rarely survive to days 5/6. Others share our opinion on this subject as well [26].

Since increasing numbers of IVF centers now, however, resort to routine blastocyst stage culture, including in women with low FOR and at advanced ages, we have had ample opportunities to treat women who failed multiple such attempts before presenting to our center. Though not a “controlled” study set up, we in a good number of such patients succeeded in establishing pregnancies (reaching normal delivery) who in repeated preceding cycles either failed to have embryos reach blastocyst stage and/or failed to reach embryo transfer for other reasons [27] and Gleicher N, unpublished data. This is also a reason why we oppose PGS in older women, when used to improve IVF outcomes [27]. PGS in older patients actually, for all of above noted reasons, appears to reduce pregnancy chances in association with IVF [28].

### Systematic literature review and discussion

#### National U.S. aging trends

**Figure 2:**
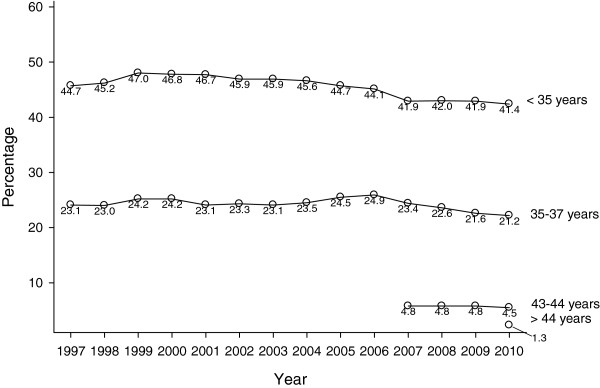
**Percentage distribution of U.S. IVF cycles per age groups, years 1997-2010.** The figure demonstrates in the young patient groups (<35 years and 35-37 years) flat IVF cycle years for the period between 1997-2010, and even mild declines, starting in 2006/2006. In contrast, the oldest patient groups, before 2007 not even registered in national outcome reporting in the U.S., gathered steam. Starting in 2010, national outcome reporting for the first time, indeed, included women above age 44 years. All of these development also correlate well to reported U.S. data, suggesting that women above age 40 now represent the, proportionally, most rapidly age group of women having children [1,2].

Figure 2 summarizes national U.S. age distributions for IVF, reported to the CDC between 1997 and 2010 (Center for Disease Control and Prevention 2013). Till 2006 CDC published patient outcome data only up to age 42 years; since 2007 up to age 44 and, starting in 2010, also including women above age 44. In 1997 women under age 35 represented 44.7 percent of all fresh non-donor IVF cycles; by 2010, however, only 41.4 percent (Table 2 and Figure 2). Concomitantly, as further evidence for the “graying” of fertility care in the U.S., the number of donor egg cycles more than doubled between 1997 and 2010 (Table 2). Growth in donor egg cycles at our center also exceeded growth in fresh IVF cycles over the last five years (data not shown).

**Table 2:** Age distribution of U.S. IVF cycles 1997-2010

	**Total**	**<35**	**35-37**	**38-40**	**41-42**	**43-44**	**>44**	**Donor ETs**
1997	55,042	24,581 (44.7%)	12,733 (23.1%)	10,997 (20.0%)	6,691 (12.2%)			4,498
1998	61,650	27,858 (45.2%)	14,146 (23.0%)	12,037 (19.5%)	7,609 (12.3%)			5,308
1999	63,123	29,682 (47.0%)	15,291 (24.2%)	12,848 (20.4%)	5,302 (8.4%)			5,844
2000	71,556	33,453 (46.8%)	17,284 (24.2%)	14,701 (20.6%)	6,118 (8.6%)			6,731
2001	77,102	35,984 (46.7%)	17,791 (23.1%)	16,283 (21.1%)	7,044 (9.1%)			7,722
2002	81,888	37,591 (45.9%)	19,110 (23.3%)	17,454 (21.3%)	7,733 (9.4%)			8,394
2003	86,753	39,852 (45.9%)	20,056 (23.1%)	18,660 (21.5%)	8,185 (9.4%)			8,970
2004	89,535	40,853 (45.6%)	21,019 (23.5%)	19,174 (21.4%)	8,487 (9.5%)			9,283
2005	92,405	41,302 (44.7%)	22,624 (24.5%)	19,482 (21.1%)	8,997 (9.7%)			9,649
2006	93,866	41,369 (44.1%)	23,376 (24.9%)	19,775 (21.1%)	9,346 (10.0%)			10,049
2007	100,592	42,127 (41.9%)	23,504 (23.4%)	20,612 (20.5%)	9,535 (9.5%)	4,814 (4.8%)		10,321
2008	103,104	43,296 (42.0%)	23,326 (22.6%)	21,793 (21.1%)	9,783 (9.5%)	4,907 (4.8%)		10,718
2009	101,090	42,384 (41.9%)	21,860 (21.6%)	22,144 (21.9%)	9,845 (9.7%)	4,857 (4.8%)		10,151
2010	100,824	41,744 (41.4%)	21,369 (21.2%)	21,741 (21.6%)	10,122 (10.0%)	4,501 (4.5%)	1,347 (1.3%)	9,866

As women age, they require earlier utilization of IVF and higher medication dosages for ovarian stimulation. Increasing embryo aneuploidy with advancing maternal age [29], and increasing miscarriage risk [30], together with higher medication costs, lead to higher treatment costs per cycle, while efficacy of treatments declines in parallel [30]. Cost effectiveness of IVF, therefore, decreases with advancing female age (see also later).

Practically all infertility treatments convert natural, mono- follicular into poly-follicular cycles, increasing the risk of multiple births [31]. Older maternal age during 1980-2009 accounted for approximately one-third of the U.S. increase in twinning [32]. Bamberg et al. reported 34.4 percent of twin pregnancies to be due to infertility treatments, a 3.2-year increase in maternal mean age over their study period, more infertile women above age 35 than among spontaneously conceived twins (37.6% vs. 22.9%), and significantly higher mean ages (32.5 vs. 30.1 years) [33].

This development is at least partially driven by advancing female age and increasing length of infertility increasing the desire for multiple births [34]. The increasing complexities encountered by older women pursuing pregnancies and entering fertility care, therefore, are multifactorial (see also later).

### Limited access to care

The increase in older women pursuing pregnancy is taking place against a background of considerable skepticism and even resistance from the U.S. government, the insurance industry and many colleagues in the medical community [30,35,36].

In many European countries advanced age is often considered a categorical barrier to treatment. For example, in the UK coverage is limited to ages 23-39 years [37], though an expansion to age 42 has recently been proposed [38]. Sweden restricts access to fertility treatment after age 40-42 years [39], and Finland after age 40 years [40].

Cross-border medical tourism in search of fertility services has, therefore, greatly increased amongst older patients [41,42]. Our own center has witnessed a remarkable increased in older patients from Scandinavian countries over recent years, Particularly older Swedish patients in their country appear to face almost complete exclusion from access to fertility treatments, including from private centers. Prohibition of oocyte donation in many countries further exacerbates need for travel to receive fertility care [41].

In the U.S., restrictions are often more subtle. Medicaid and Medicare, the two federally funded government health care programs, do not offer coverage for IVF. The effects of the Affordable Care Act (“Obamacare”) on IVF coverage are as of this point unpredictable, as is the future of the whole program, In the private insurance market age-linked restrictions greatly vary even within different insurance plans offered by the same insurance companies.

The medical profession, however, also contributes to some of the resistance in Europe and the U.S. Most fertility centers on both sides of the Atlantic maintain rigid age-cut offs and/or link denials of treatment to laboratory parameters, reflective of FOR. Indirectly, these laboratory parameters, of course, also reflect age [43], though patients may be unaware that, for all practical purposes, age-associated cut-offs are applied.

Our review of available data suggests that such age-associated restrictions to treatment access lack basis in evidence since other factors than age also play major roles in determining pregnancy chances. Indeed what pregnancy chances are is often incorrectly assessed. For example, we find that patients frequently are advised that above age 40 years pregnancy chances from IVF and intrauterine inseminations (IUI) are similar. They, therefore, are refused IVF, and instead offered IUI cycles. Since 2001 the literature, however, suggests that IVF actually offers clearly superior pregnancy chances and time to conception at older ages in comparison to IUIs [44,45].

Many colleagues also routinely advise patients that, above age 40 - 42 years, IVF live birth rates are in the 1-2% range. As here presented data demonstrate, such a claim is, however, likely at least up to and inclusive of age 45 years unsupported, utilizing contemporary IVF standards of care, especially if patients are willing to consider consecutive cycles [5] and Table 1. Where this widely shared opinion originates is, therefore, unclear.

The most frequent reason for withholding infertility treatments amongst colleagues has been concern about cost-effectiveness of such treatments in older women [43,46]. Other motives, however, can also play a role: For example, since fertility centers often compete based on clinical pregnancy and delivery rates, lower pregnancy chances of older women can drag down a center’s overall IVF outcomes. In the U.S. such considerations are condemned by professional guidelines [8].

The obvious economic importance of pregnancy and delivery rates for IVF center was recently, however, again demonstrated when a small group of U.S. centers was reported to manipulate their contributions to the national outcome reporting system [47] by excluding unfavorable patients from reporting, either cancelling cycles before retrieval or avoiding reportable embryo transfers by cryopreserving all embryos without attempting a transfer [48].

Motivations for cycle cancellations can also contribute to outcome reporting biases: Some centers maintain unrealistic minimum follicle numbers for taking a patient to egg retrieval; others artificially increase cycle cancellations by routinely culturing embryos of older women’s to day-5/6 blastocyst-stage instead of transferring on day-3 [26]. A recently increasingly propagated protocol adds preimplantation genetic screening (PGS) after trophectoderm biopsy to routine IVF [49]. In older women only few embryos, however, usually survive to blastocyst stage, and even fewer will be euploid. So treated older women will, therefore, only infrequently reach embryo transfer and, therefore, escape reporting requirements [26,27,48]. Reported IVF pregnancy and delivery rates in such cases, therefore, are misleading since they are not calculated by “intent to treat” (i.e., cycle start) but use as reference point embryo transfer, which older women, of course, only rarely achieve.

### When should treatment be considered futile?

For colleagues concerned about cost effectiveness of IVF at advanced patient ages, the central question becomes at what point treatments should be considered futile. The *Ethics Committee of the ASRM* defines “futility” as equal to or less than a one-percent chance of live birth. A “very poor” prognosis, in contrast, is defined by low but not nonexistent chances of live births (>1% to ≤5% per treatment cycle) [8].

Most colleagues providing fertility services on both sides of the Atlantic, currently, likely, consider almost all women above age 40-42 to fall into these two patient categories. Under guidelines from the *Ethics Committee of the ASRM*, physicians, therefore, may under such circumstances refuse treatment of patients. These guidelines, however, also recommend that, in case of treatment refusal, such patients be referred to providers who do offer treatment to older patients. This, however, rarely, if ever, happens because affected patients usually are advised that their only chance of pregnancy is with help of donor oocytes. As already noted before, the literature would suggest that this is incorrect advice [5] and Table 1, and many patients consider oocyte donation only a second-best choice.

### Reported clinical outcome data in older women

Our search failed to locate convincing evidence for reliable age-specific IVF outcome reports above age 40-42. Table 3 summarizes limited published data on the subject: Spandorfer et al. reported on 288 women above age 44 years (mean 45.4 ± 0.73). Only 161 among them reached retrieval (clinical pregnancy rate 21.2%, miscarriage rate 85.3%), resulting in a disappointing live birth rate of only 3.1% [50].

**Table 3:** Reported pregnancy rates in infertile women above age 40

**Author**	**IUI/IVF**	**% pregnancies**	**% live births**	**Year of reference**
***Spandorfer et al.***^***1***^[50]	IVF	21.2	3.1	2007
***Tsafrir et al.*** [45]	IUI/IVF			2009
***Age 40***		13.9	9.1	
***Age 45***		2.8	0.7	
***Schimberni et al.*** [51]	IVF			2009
***Per cycle***		5.8		
***Per transfer***		10.5		
***Hourvitz et al.*** [35]	IVF			2009
***Age 42***		7.7		
***Age 43***		5.4		
***Age 44***		1.9		
***Marinakis and Nikolaou*** [36]	IVF			2011
***Age 40-42***			11.0	
***Age 43-44***			4.6	
***Age >44***			< 4.0	
***Ninimäki et al. ***^***2***^[7]	IVF			2012
***1 embryo ET***		19.5	11.0	
***2 embryo ET***		23.5	13.6	

^1^After reviewing the literature, concluded that after age 40-41 IVF was preferable to IUI;

^2^Reflects patients between ages 40-44 years;

ET, embryo transfer.

Tsafrir et al. reported pregnancy and delivery rates of 13.9% and 9.1% at age 40, and 2.8% and 0.7% by age 45 years [45], Italian investigators reported a clinical pregnancy rate of 5.8% in women 40 or older per cycle start, and 10.5% per transfer [51]. Hourvitz et al. reported a clinical pregnancy rates per cycle start of 7.7% at age 42, 5.4% at age 43, and 1.9% at age 44, concluding that IVF should be restricted to under age 43 years [35]. As noted before, reviewing the literature Tsafrir et al. concluded that IVF, despite low pregnancy rates (<5%), was preferable to IUI in women above age 40-41 [45].

In 2011 professional societies in Canada published a whitepaper, which, without quoting pregnancy expectations, noted that above age 40 IVF should be considered after only one or two failed cycles of controlled ovarian stimulation [52].

Marinakis and Nikolaou reported live births rates in the United Kingdom (UK) of 11.0% at ages 40-42, 4.6% at ages 43-44 and less than 4.0% above age 44 [36]. Soullier et al. reported delivery rates of 4.0 percent for women above age 40 [53]. Analyzing 124,148 IVF cycles (33,514 live births) in the U.K., Lawlor and Nelson reported that two embryos transfers increased odds of live births at ages 40 or older more than in younger women, demonstrating that outcomes in older women can be improved by increasing embryo numbers transferred [54]. Ninimäki et al. were outliers in their outcomes, reporting with transfer of two and one embryo, respectively, pregnancy rates of 23.5 and 19.5 percent and live birth rates of 13,6 and 11.0 percent between ages 40-44 [7].

Skepticism towards treatment of older women is understandable, considering such limited and generally low reported outcome data. Lack of evidence in favor of treatment should, however, not be misconstrued as evidence in favor of no treatment.

Available data, therefore, have to be analyzed with caution. They suggest that: (i) only few centers worldwide routinely treat older women to IVF cycle completion; (ii) older patients, therefore, frequently are not given the opportunity to benefit from current state-of-the-art IVF treatments; Consequently, (iii) available outcome data are insufficient. Maybe most importantly, however, (iv) absence of controlled treatment attempts of older women with use of their own oocytes prevents improvements in treatment outcomes of such patients, creating a vicious circle to the detriment of older women,

### Patient autonomy

Agreeing with a recently voiced ethical opinion by French colleagues in association with IVF [55], our center advocates patient autonomy in all decision-making processes in association with IVF. We fully support the deliberative, case-by-case approach, advocated by these authors in allowing patients to reach informed decisions. As part of this process we see it as our responsibility as physicians, at all stages, to (i) inform patients in unbiased form about their options, and (ii) advise patients, based on our own center’s outcome data, what their chances of treatment success/failure are with each treatment option. We then defer to the patients’ decisions, as long as they do not unreasonably endanger their own wellbeing or that of their potential offspring(s). Above age 45 years, the process, therefore, involves extensive medical as well as psychosocial evaluations of patients.

This approach makes infertility treatments at our center, in principle, available to almost all patients who are not in menopause (FSH ≥ 40.0 mIU), and explains the extreme adverse selection of our center’s patient population, described earlier.

Patient autonomy also deserves consideration wen women are emotionally not ready to proceed into oocyte donation. Often they, first, have to convince themselves that they have exerted maximal efforts with use of their own eggs. Advising such women that oocyte donation represents their only reasonable chance of pregnancy is, therefore, often not enough. They frequently require additional cycle attempts with their own ovaries before reaching a point of conviction that allows them to proceed with donor eggs.

We consider it appropriate to offer these opportunities, since women who are prematurely “forced” into egg donation for the rest of their lives may second-guess their decision, even if they successfully conceived and delivered. Indeed, the newborn child may become the source of this second-guessing, which in rare instances can lead to significant psychological complications in the mother/child relationship, even rejection of the child by the mother (Gleicher N, unpublished data).

### Cost-effectiveness

As noted earlier, cost-effectiveness is often the principal argument against treating older patients [30]. Paradoxically, this is an argument most prevalent in countries perceived as “social” in political outlook, like, for example, in the Scandinavian countries, These countries often do not consider expenditures on fertility treatments in older women to meet minimum thresholds of cost effectiveness [40]. Insurance companies in the United States (U.S.) have in some states voiced similar arguments in support of age restrictions in coverage of fertility services or in opposition to mandated insurance coverage for fertility services [56,57].

Age-dependent rationing of medical care is a widely accepted concept in many European countries [40], while fear of such rationing in the U.S. has been a major reason for opposition to the recently passed Affordable Care Act (“Obamacare”) [58]. Defining cost-effectiveness of infertility care is, therefore, as much an economic as a political issue, often as much affected by geopolitical considerations as by pure cost considerations.

The state of Israel is a good example: With the highest utilization of IVF in the world (1657 IVF cycles/million citizens/year), this small country performs almost twice the number of IVF cycles of Iceland, the second highest utilizing nation [59]. The reason is that the Israeli government considers any subsidy of IVF, at almost all ages, “cost-effective” because population growth is considered essential to its economic development and national security [60].

This can be contrasted with the Canadian province of Québec, where the state government only agreed to assume costs for IVF coverage in return for a commitment by the local provider community to reduce twin pregnancies by accepting a single embryo transfer (e-SET) mandate [61]. Québec and Israel’s governments, thus, very obviously, reached very different “cost-effectiveness” conclusions. Aside from remaining questions whether twin pregnancies really increase health care costs, considering the loss of life-long economic benefits from “lost” births in Québec, one has to wonder whether the province’s decision, indeed, can be considered “cost-effective” [62].

Objective assessments of cost-effectiveness are further complicated by the greatly varying methods in how cost-effectiveness is assessed in different countries. In The Netherlands, Evers, for example, calculated the lifelong contribution of every newborn to the growth national product (GNP) at €1,848,320, while societal costs, including childcare, education social welfare and health care costs add up to only €1,610,000. He concluded that every birth leaves Dutch society with a net-gain of approximately €238,320 (US$ ca. 303,000), in his opinion rendering the funding of IVF up to age 44 years cost effective [63]. We are unaware of similar studies in other countries, and Evers’ calculations for The Netherlands are, of course, not universally applicable.

### Further legal and ethical considerations

Whether older women should be afforded a chance of conceiving is also a question with significant legal and ethical dimensions. Achieving motherhood represents fulfillment of a most basic human need (Perla L [64]; Smajdor A, [65]). Not to consider this fact, even in associations with cost-effectiveness considerations, therefore, appears inhumane.

The concept of universal reproductive rights is based on the recognition that individuals have the absolute right to decide freely and responsibly about number, spacing and timing of their children, free of discrimination (which includes age-discrimination), coercion and violence (Gender and reproductive rights home page, 2013).

Like other patients, older women are entitled to ethical treatment, including autonomy (of decision making), beneficence, non-maleficence and justice. A number of ethicists have addressed the desire of older women to conceive: Perla, emphasizes respect for personal patient autonomy and staff empathy [64]. Smajdor notes that, with IVF representing medical treatment, it would be unethical to use it as a means of social control, providing or withholding it on basis of moral judgments about a patient’s values or her lifestyle [65].

Delaying childbearing cannot simply be explained by the public’s ignorance of appreciating the biological relationship between female aging and female ability to conceive [66]. Society, therefore, has to accept that the increase in the number of older women having children to a significant degree is caused by objective societal developments, and not the personal fancy of only a few outliers.

### Effects on pregnancy management

With advancing age women develop increasing numbers of medical disorders [67]. At least some of these conditions, for example autoimmune diseases, can affect fertility potential [68,69] and/or raise outcome risks for mothers and offspring [69-73]. Prospective risk management, therefore, becomes essential in older women to avoid preventable pregnancy losses and other complications at varying gestational stages.

In older women fertility treatments, therefore, require increased attention to confounding medical problems, not always easily apparent in routine pre-IVF evaluations. Testing requirements, therefore, increase, as do consultations from other medical specialties. Not to be forgotten are socio-economical evaluations since any desire for motherhood at advanced age needs to be matched by social and economic abilities to parent a child, and to care for the child’s upbringing.

As medical complications are more commonplace in older women, medical providers, including obstetricians, perinatologists, neonatologists and consultants from other medical specialties, have to be ready for an increasing volume of more complicated pregnancies, [70 = 73]. Increasing adverse maternal and neonatal outcomes have to be expected as a consequence.

These evolving changes have not been appreciated in full. For example, our medical specialty has largely failed to recognize the contribution of older women to the increased numbers of multiple births, mostly twins [31-33], while concentrating on IVF in criticism [74,75]. Only a very recently published study for the first time acknowledged the contribution of an aging infertility population to the multiple pregnancy issue, following fertility treatments [76].

Putting aside whether twins really represent adverse outcomes of infertility treatments [77], older women face different risk/benefit consideration than younger patients. Almost two decades ago, we for the first time reported on the strong desire of infertility patients to conceive twins, which increases with length of infertility and advancing patient age [34]. Those sentiments should not surprise, as older women have lower chances and less time to complete their families. Scotland et al. more recently in a European patient population noted that patients, therefore, are willing to take carefully considered, and educated risks to compensate for their lower pregnancy chances [78]. Can older women, therefore, really be blamed for, at times, making different risk/benefit choices than younger women?

### A brief discussion of our center’s data

We previously noted under materials and methods that our here presented outcome data in older women have to be viewed with caution because they were achieved in a highly adversely selected patient population who, almost uniformly, were on supplementation with DHEA. Such supplementation has still remained somewhat controversial in its efficacy. While it would exceed the framework of this manuscript to document the rational of this treatment approach in older women, suffice to say that our analysis of published data, relying on animal as well as human data, strongly supports supplementation of older women with androgens due to a relative hypoandrogenemia in such patients in comparison to younger ages [14,15]. A relative recent review of the subject was published, to which interested readers are referred [17,18].

## Conclusions

Reproductive medicine is inching ever closer towards technical abilities, which will allow for successful reproduction, almost independent of female age. Recent evidence that sperm and oocytes can be derived from testicular [79] and ovarian stem cells [80], and that even pluripotent adult stem cells can be used to produce gametes [81,82], will, likely, make age-independent human reproduction a clinical reality within the foreseeable future. If progress in infertility treatments over the last 10-20 years has generated a pregnancy boom for women in their 40s, independence from ovarian and testicular senescence will expand this boom into the females’ 50s and, maybe, even 60s.

Societal consequences will be highly significant: With women’s lifespans in many industrialized countries now exceeding 80 years, even 50-year old mothers now will have postmenopausal life expectancies exceeding postmenarcheal life expectancies of much younger mothers at the beginning of the 20^th^ Century. Women, therefore, will increasingly give birth to children at what, just one to two generations ago, used to be grandparental ages. Consequences will not only be medical in nature but will permeate all aspects of modern society. Against a backdrop of already budget-bursting health care costs, medical practice and society better get ready for this revolution!

## Abbreviations

AMH: Anti-Müllerian hormone; CDC: Centers for Disease Control and Prevention; DHEA: Dehydroepiandrosterone; DOR: Diminished ovarian reserve; EC-ASRM: eSET, Elective single embryo transfer; FOR: Functional ovarian reserve; FSH: Follicle stimulating hormone; HEFA: Human Fertilisation and Embryology Authority; hMG: Human menopausal gonadotropin; IUI: Intrauterine insemination; IVF: In vitro fertilization; PGS: Preimplantation genetic screening; POA: Premature ovarian aging; OPOI: Occult primary ovarian insufficiency; OR: Ovarian reserve; T: Testosterone; U.K: United Kingdom; U.S: United States.

## Competing interests

All four authors received grant support, travel funds and speaker honoraria from pharmaceutical and medical device companies, none, however, related to this manuscript. N.G. and D.H.B. are co-inventors of already awarded and still pending patents, relating to (i) supplementation of women with dehydroepiandrosterone (DHEA) and other androgens to improve conception and delivery chances, and (ii) use of *FMR1* gene mutations to assess risk towards premature ovarian aging and potential cancer-causing effects in association with *BRCA* mutations. N.G. is owner of CHR-NY, and owns shares in fertility Nutraceuticals, LLC. N.G. and D.H.B receive patent royalties from this company. N.G. and D.H.B, own shares in LifeCycle Laboratories LLC and have licensed *FMR1* patents to this company. V.A.K. serves as a consultant to the Centers for Disease Control (CDC) in regards to national ART outcome reporting. A.W. has no potential conflicts to report.

## Authors’ contributions

Study concept and design: NG Acquisition of data: NG, VAK, DHB Analysis and interpretation of data: NG, VAK, DHB. Drafting of the manuscripts: NG Critical revision of the manuscript: NG, VK, AW, DHB. All authors read and approved the final manuscript.
